# Inferring individual evaluation criteria for reaching trajectories with obstacle avoidance from EEG signals

**DOI:** 10.1038/s41598-023-47136-2

**Published:** 2023-11-17

**Authors:** Fumiaki Iwane, Aude Billard, José del R. Millán

**Affiliations:** 1 Learning Algorithms and Systems Laboratory (LASA), École Polytechnique Fédérale de Lausanne (EPFL), 1015 Lausanne, Switzerland; 2 Chandra Family Department of Electrical and Computer Engineering, The University of Texas at Austin, Austin, TX 78712 USA; 3 Department of Neurology, The University of Texas at Austin, Austin, TX 78712 USA; 4 Department of Biomedical Engineering, The University of Texas at Austin, Austin, TX 78712 USA; 5 Mulva Clinic for the Neurosciences, The University of Texas at Austin, Austin, TX 78712 USA

**Keywords:** Computational neuroscience, Biomedical engineering, Perception

## Abstract

During reaching actions, the human central nerve system (CNS) generates the trajectories that optimize effort and time. When there is an obstacle in the path, we make sure that our arm passes the obstacle with a sufficient margin. This comfort margin varies between individuals. When passing a fragile object, risk-averse individuals may adopt a larger margin by following the longer path than risk-prone people do. However, it is not known whether this variation is associated with a personalized cost function used for the individual optimal control policies and how it is represented in our brain activity. This study investigates whether such individual variations in evaluation criteria during reaching results from differentiated weighting given to energy minimization versus comfort, and monitors brain error-related potentials (ErrPs) evoked when subjects observe a robot moving dangerously close to a fragile object. Seventeen healthy participants monitored a robot performing safe, daring and unsafe trajectories around a wine glass. Each participant displayed distinct evaluation criteria on the energy efficiency and comfort of robot trajectories. The ErrP-BCI outputs successfully inferred such individual variation. This study suggests that ErrPs could be used in conjunction with an optimal control approach to identify the personalized cost used by CNS. It further opens new avenues for the use of brain-evoked potential to train assistive robotic devices through the use of neuroprosthetic interfaces.

## Introduction

We use our arm to reach out for objects of all sorts during daily activities. As we do so, we make sure not to intersect with obstacles on the way. Research on human motor control aims to understand the factors driving reaching motion with a general consensus that the dynamics of these movements is driven by an internal model^1^. In general, reaching paths are straight with a bell-shaped velocity profile, as the central nervous system (CNS) optimizes the effort and time of the reaching actions^2,3^. The optimal control view explains a wealth of evidence on how the CNS can modulate the trajectory online to adapt to environmental constraints, e.g., obstacle avoidance^4^. However, experimental evidence suggests that several factors are at play when generating trajectories and that these factors vary across individuals^5^. Efficiency and comfort are two key factors when computing reaching motion, where one aims at a good balance between optimizing for efficiency, by minimizing effort^3,6^, and maximizing (arm) comfort, e.g., by steering away from the joint limit^7,8^. These two factors often conflict, such as when reaching for a target while avoiding obstacles. Taking the shortest path may require to bring the arm dangerously close to the obstacle, and require uncomfortable stiffening of the muscles to prevent inadvertent contact^9^.

Reaching trajectories follow a similar pattern, but there exists variability between individuals^5,10^. Individuals may opt to modify their natural arm trajectory to reduce risks, choosing a longer, and hence less efficient path^11^. Although a previous study^5^ characterized such individual variation in reaching trajectories, it is not known if this variation is associated with a personalized cost function used for the individual optimal control policies^4^ and how it is represented in our brain activity. To address this question, we investigate whether such individual variations in their desirable reaching trajectories are reflected in the two parameters of the control policy, energy efficiency and comfort, and if it is possible to infer individual evaluation criteria from their electroencephalogram (EEG) activity. We therefore hypothesize that the personalized evaluation criteria are revealed in a differentiated weighting given to energy minimization versus comfort. To maintain a consistent viewpoint across individuals, we generate human-like reaching trajectories and assume that such preferences can be revealed not only when producing self-generated reaching movement, but also when observing others’ reaching movements^12,13^, even when these are generated by artifacts such as robots^14^. Furthermore, we monitor brain error-related potentials (ErrP)^15–17^, which may be elicited when subjects observe a robot moving around a fragile object, displaying at times daring trajectories around the object.

ErrPs have been used in brain-machine interface (BMI) scenarios to infer the user’s perception of correctness of actions executed by the machine. In many cases, the machine was a robotic system: either a robot arm^18–20^ or a vehicle^21^. To characterize one’s evaluation criteria with ErrPs, participants need to monitor a vast amount of systematically modulated human-like reaching trajectories. For example, Kolkhorst *et al* showed recorded videos of two different robot trajectories to participants and used elicited ErrPs to evaluate these trajectories^22^. This approach, however, is limited both on the number of trajectories and on the way that the trajectories were evaluated —the ErrPs were extracted when the participants listened to statements describing the robot motion (e.g., ‘Path 1 is better than Path 2’).

This present study extends the analysis of the data collected in our previous study^23^, in which subjects observed the motion of a robot arm passing close-by a fragile obstacle, namely a wine glass. Subjects were in partial control of the robot as they could initiate the robot’s motion or interrupt it when they deem the robot to come too close to the obstacle. Outside these human interventions, the robot was fully autonomous, controlled by dynamical systems.

Dynamical systems are sets of ordinary differential equations. They have been shown to account for the dynamics of human reaching movements^24–26^ and the curvature of reaching movements in 3D and higher dimensions^27,28^ in constrained environments^29^. Coupling across dynamical systems has been used to model limb coordination in reaching, such as coordination between the grip aperture and arm motion^30,31^, and the coordination across the visual and motor systems to avoid obstacles^32^.

**Figure 1:**
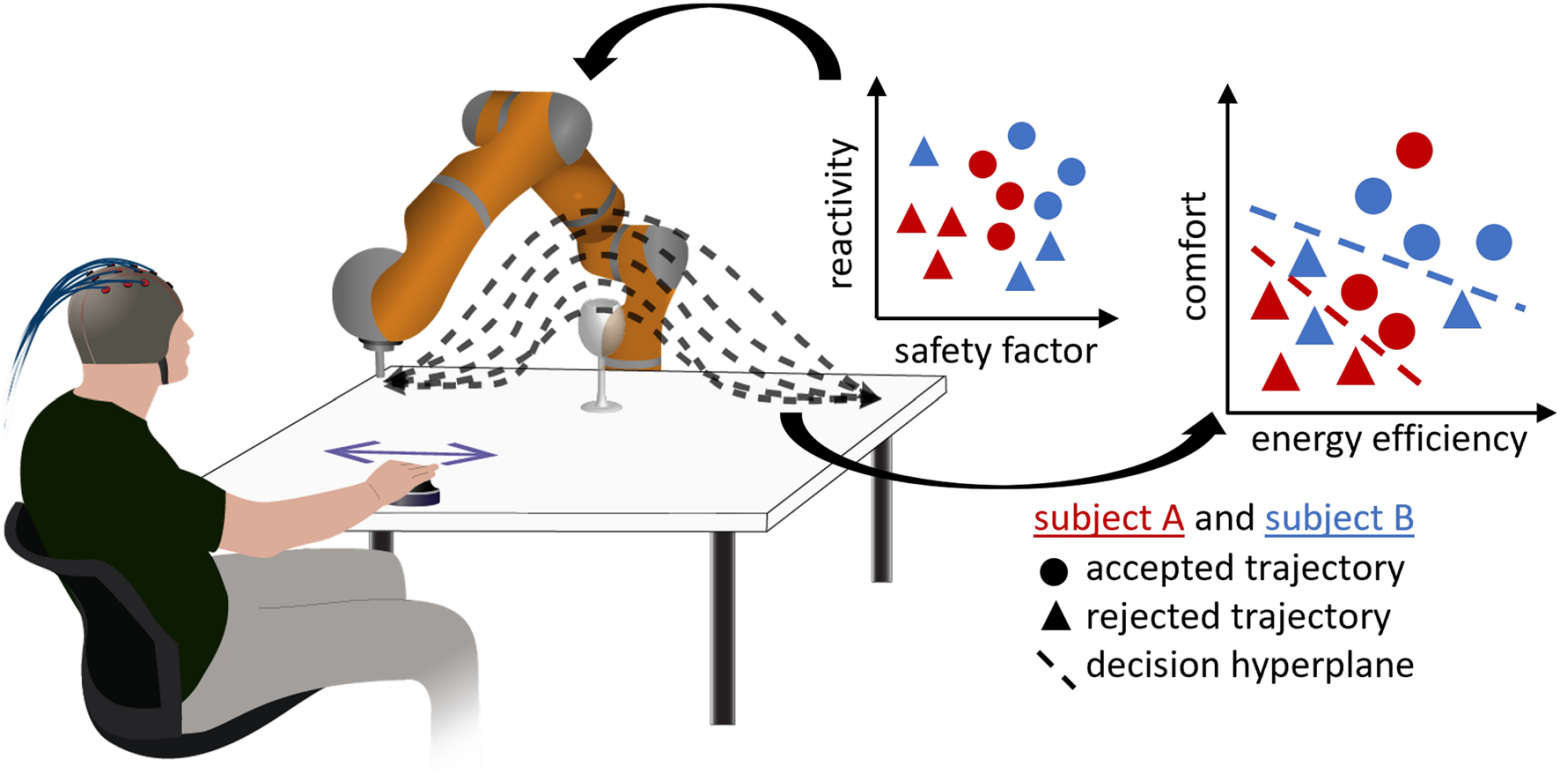
Experimental protocol. Participants directed the robot by using the joystick in their hand and evaluated a variety of robot trajectories with obstacle avoidance while collecting their EEG signals. Robot trajectories were generated by a dynamical system and modulated by two parameters, a *safety factor* *s* to control the distance to the obstacle and a *reactivity factor* $\rho$ to determine when the robot starts avoiding the obstacle. For each robot trajectory, we compute the energy efficiency, measured as the distance travelled by the robot, and the comfort, measured as the minimum distance between the end-effector and the obstacle, and relate this to individual specific preferences. We assess such personalized evaluation criteria by transferring individually calibrated classifier across participants.

**Figure 2:**
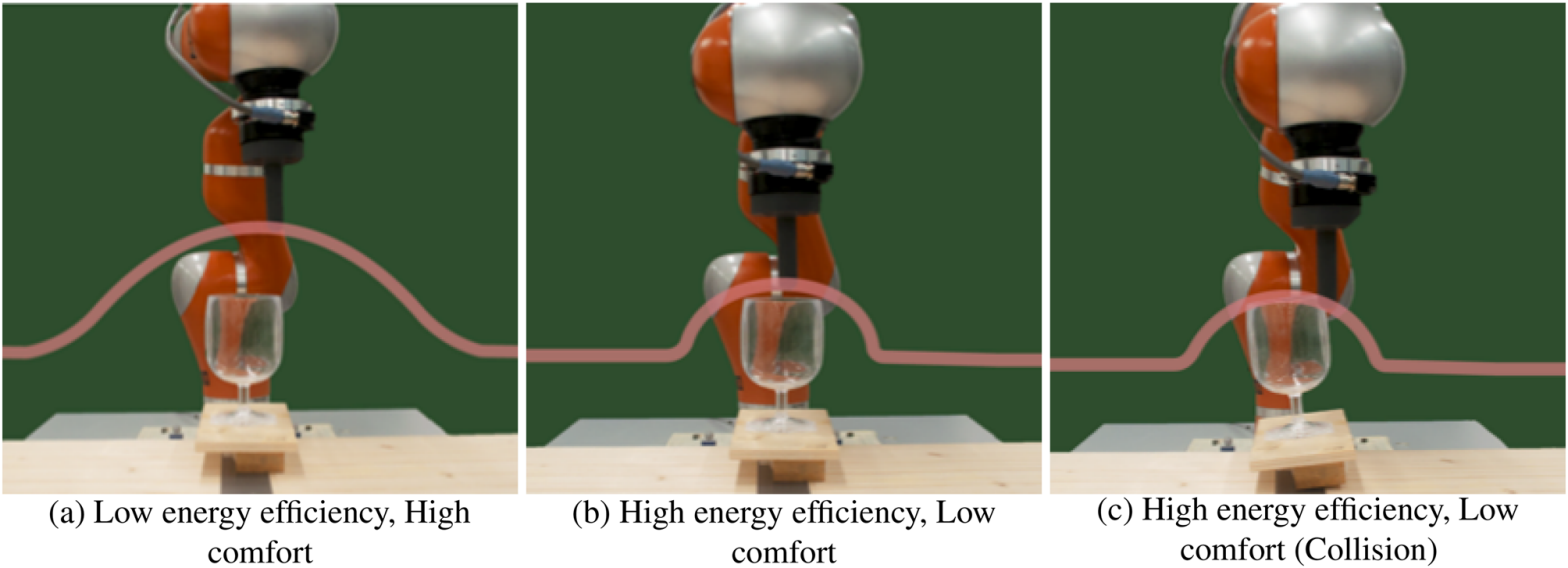
Example robot trajectories. Sets of example robot trajectories with (**a**) low energy efficiency and high comfort, and (**b**, **c**) high energy efficiency and low comfort.

Avoiding obstacles requires to continuously monitor both the arm posture and its relation to the surrounding environment (i.e., position of arm, hand, target, and obstacles). Humans seamlessly adjust the reaching trajectories upon perception of potential collisions^10^. By setting our robot’s trajectory according to a human-like dynamical system, we aimed at increasing the naturalness of the motion and hence the chance to elicit emphatic response in the observer. To systematically modulate robot trajectories, we acted on two parameters that could modify the trajectory in the vicinity of the obstacles; safety factor and reactivity (Figs. 1 and  2). The safety factor controlled the margin between the end-effector of the robot and the obstacle, while the reactivity corresponded to the point of the trajectory, with respect to the location of the obstacle, where the robot started to move away from the obstacle.

In this work, we revisit the data acquired in our previous study^23^ and investigate if the individual evaluation criteria can be attributed to different weighting in “energy efficiency” versus “comfort”. We further investigate whether ErrPs infer individual weighting of energy efficiency and comfort while evaluating human-like reaching trajectories.

## Results

### Electrophysiological results

**Figure 3:**
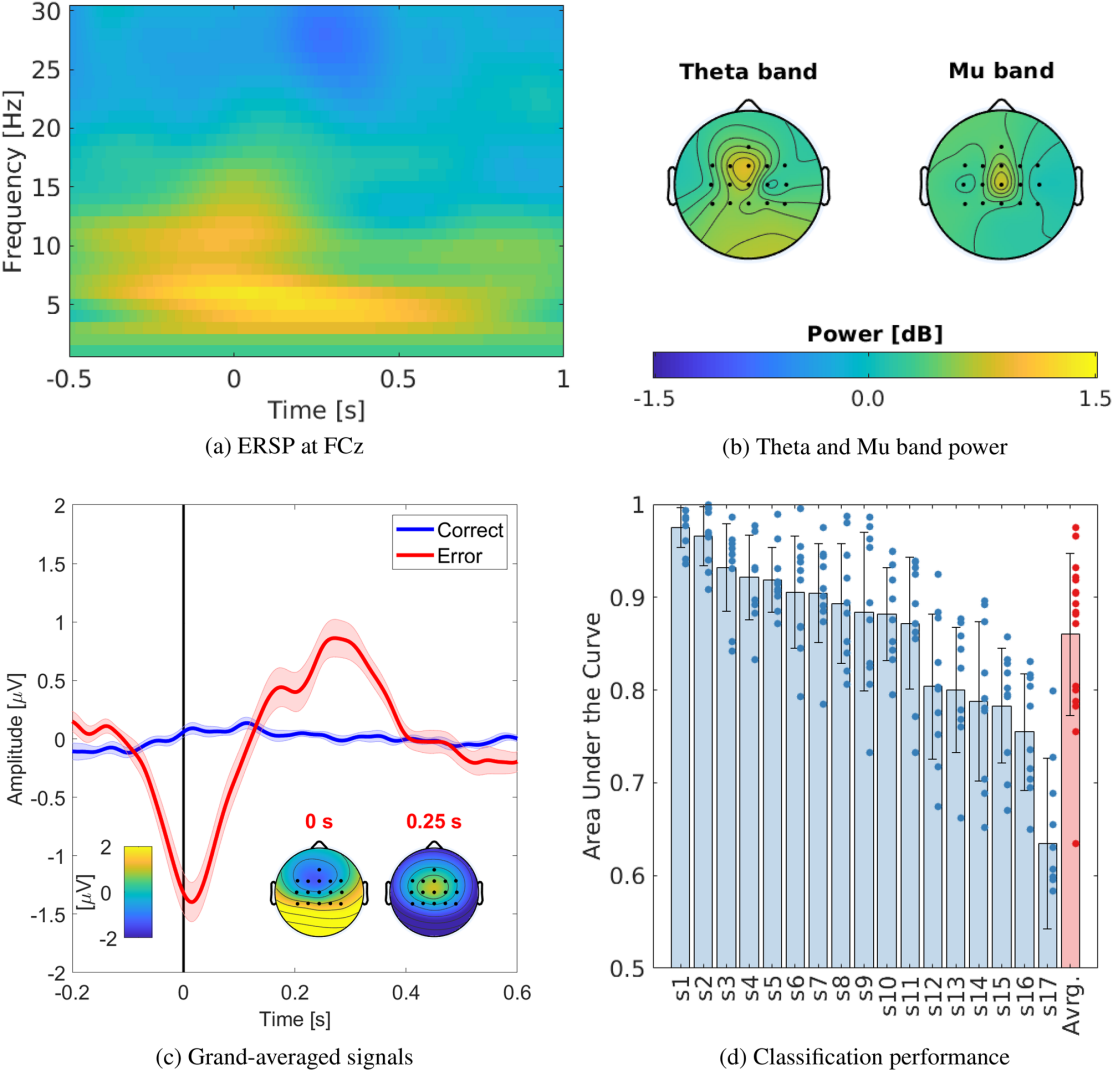
Electrophysiological results. (**a**) Time-frequency representation of error-related spectral perturbation (ERSP) at FCz within the time window of [$-0.5, 1$] s with respect to onset of joystick release. (**b**) Theta band ([4 8] Hz) and mu band ([8 12] Hz) spectral power of all channels relative to the correct trials. (**c**) Grand-averaged event-related potentials of correct and erroneous trials at FCz. 0 s in x axis represents the onset of release for erroneous trials, while it corresponds to the individual averaged release time for correct trials. Insets illustrate scalp topographical representation of the obtained ErrPs at 0 and 0.25 s. (**d**) Classification performance measured by Area Under the Curve (AUC) for each subject. Each bar corresponds to the averaged AUC, while each dot corresponds to the AUC from one testing fold or averaged AUC score of single participants (in red).

In time-frequency representations, we observed an increase of theta ([4 8] Hz) and mu band ([8 12] Hz) power in erroneous trials over the frontocentral areas; i.e., FCz channel (Fig. 3a and b)^33–38^. Theta band power increased from -0.1 s to 0.4 s, while mu band power did from -0.1 and 0.1 s relative to the release of the joystick. In the temporal domain, a clear negative deflection, ERN^16,39,40^, followed by positive deflection, Pe^41,42^, appeared over frontocentral areas (e.g., FCz electrode) for erroneous trials (Fig. 3c and Fig. S1)^17,43^. In contrast, the time-frequency representations and grand averaged signals of the correct trials remained mostly flat (Fig. S2 and Fig. 3c). Our decoding approach successfully classified the ErrPs at $0.86 \pm 0.088$ (mean ± SD) as measured by the Area Under the ROC Curve (AUC) (Fig. 3d)^13,13,44–47^. The decoding performance varied across participants due to the variability in their individual ErrPs (Fig. S1).

### Group-level comparison of behavioral- and inferred evaluation criteria

**Figure 4:**
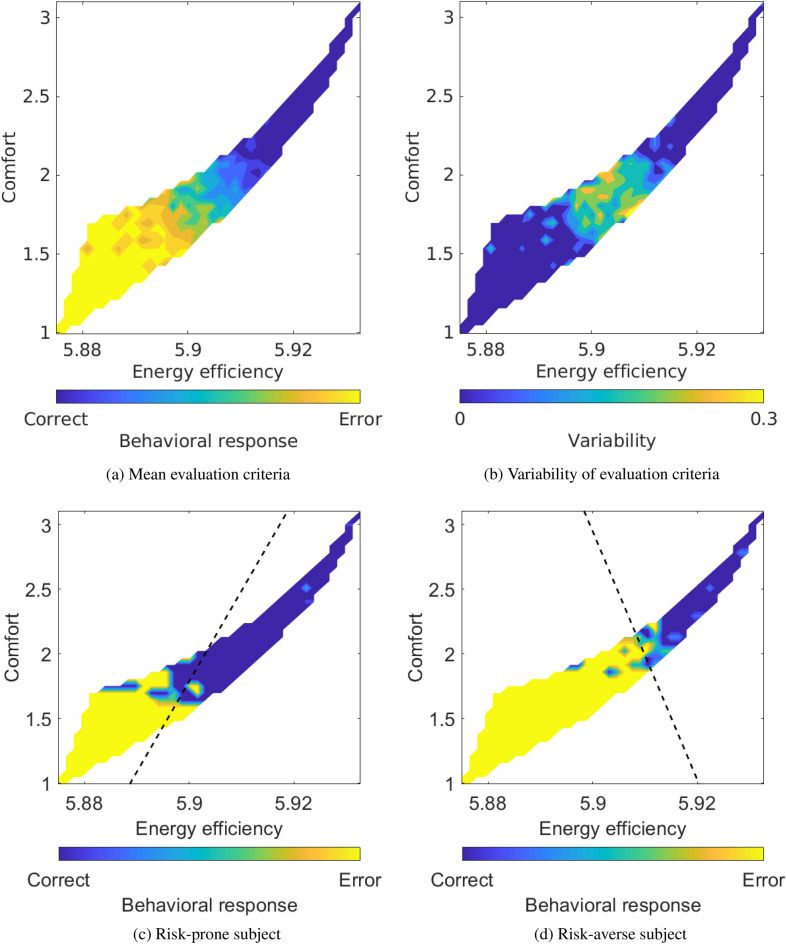
Evaluation criteria with behavioral responses. (**a**) Mean and (**b**) variability of energy efficiency by comfort matrices based on behavioral responses. (**c**, **d**) Evaluation criteria of risk-prone and -averse subjects, respectively. A dashed black line shows the decision boundary between correct and error trials.

Participants consistently released the joystick, perceiving the trajectories as erroneous, for robot trajectories being energy efficient but of low comfort (Fig. 4a). On the contrary, robot trajectories with high comfort but energy inefficient were considered correct. High variability over participants was observed around the boundary of correct and erroneous trajectories (Fig. 4b). Energy efficiency ($91.9 \pm 3.1\%$, mean ± std.) better predicted behavioral responses compared to comfort ($90.0 \pm 3.8\%$, paired Wilcoxon’s signrank test, $p=0.001$). These results illustrate that individual evaluation criteria varied around the border between correct and erroneous trajectories. On the other hand, consistent behavioral responses were observed at energy inefficient and large comfort, as well as energy efficient and low comfort.

**Figure 5:**
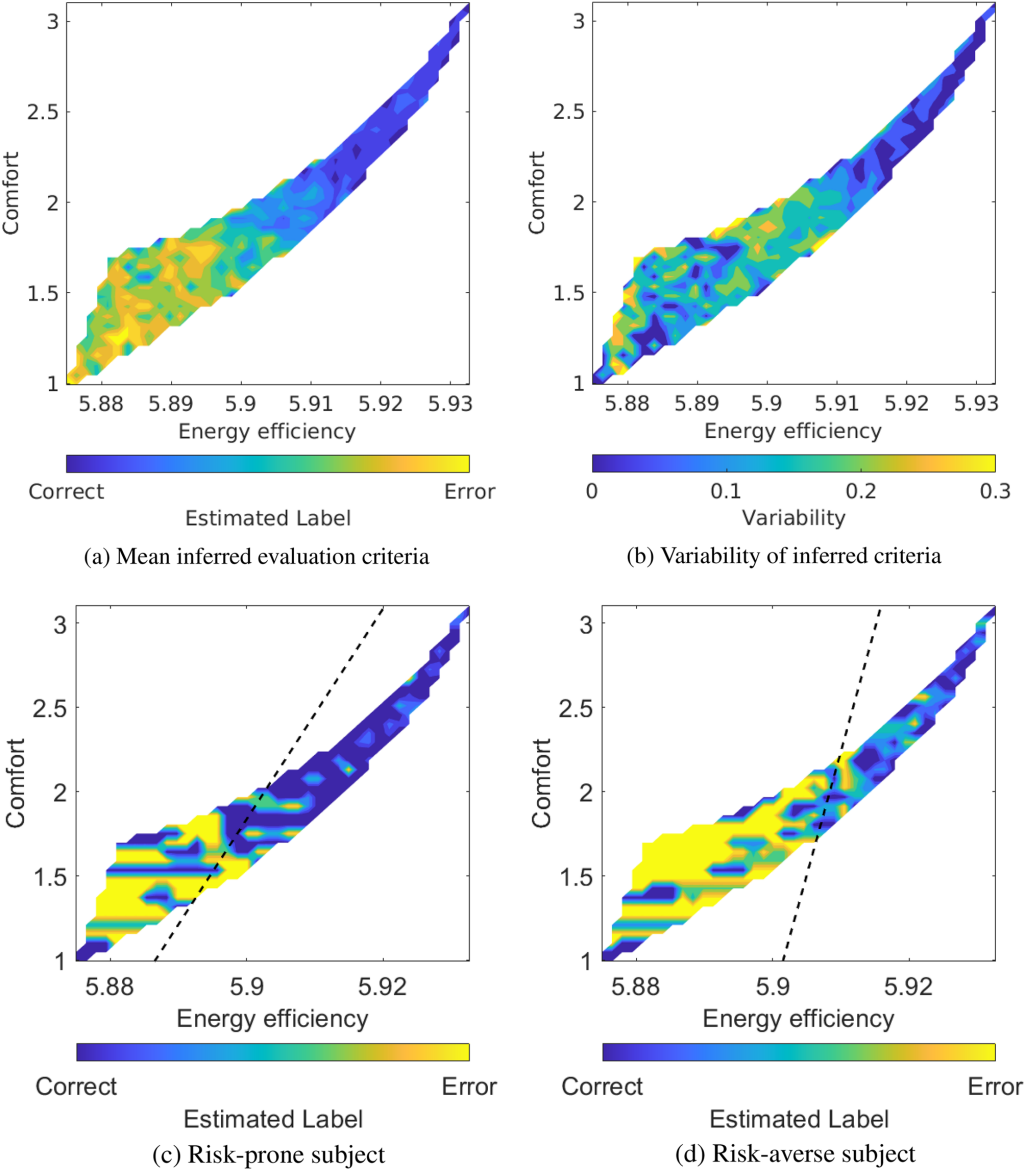
Inferred evaluation criteria based on ErrP. (**a**) Mean and (**b**) variability of energy efficiency by comfort matrices based on ErrP decoding output. (**c**, **d**) Inferred evaluation criteria based on ErrP detection for risk-prone and -averse subjects, respectively. A dashed black line shows the decision boundary between correct and error trials.

We then inferred individual evaluation criteria using the ErrP-BCI outputs (Fig. 5a and b) and compared them with those based on behavioral responses. ErrPs were detected when participants observed that the trajectories were energy efficient but of low comfort. On the contrary, ErrPs were not detected for robot trajectories with high comfort and energy inefficiency. Energy efficiency ($76.2 \pm 7.0\%$, mean ± std.) better predicted ErrP-BCI outputs compared to comfort ($72.7 \pm 7.1\%$, paired Wilcoxon’s signrank test, $p<0.001$). The mean and variability of inferred evaluation criteria co-varied with those with the behavioral responses (Pearson’s $r = 0.966, p < 0.001$ for mean, $r = 0.501, p < 0.001$ for variability) (Figs. 4a, b and  5a, b). These results indicate that the inferred evaluation criteria successfully reproduced the behavioral evaluation criteria at the group level. Importantly, the decision boundaries of the inferred evaluation criteria successfully encoded their individual variations between risk-prone and risk-averse participants (Figs. 4c,d and  5c,d).

### Assessment of individual variations in evaluation criteria

**Figure 6:**
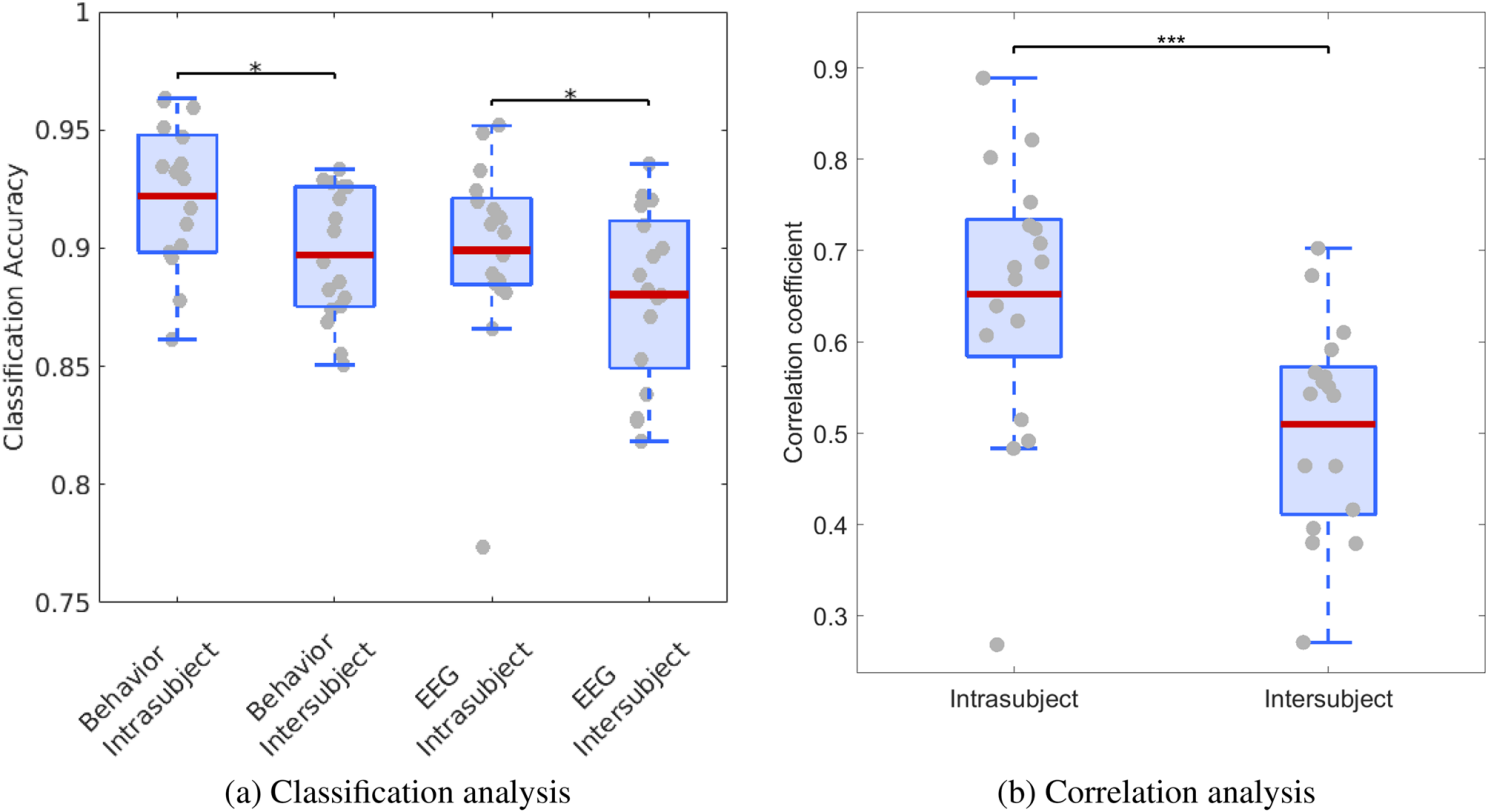
Individual customization of evaluation criteria. (**a**) Results of transfering the individually calibrated classifier between intra- and inter-subject data for two teaching labels; i.e., behavior and ErrP. $$^*$$ indicates $$p < 0.05$$. (**b**) Correlation coefficient between evaluation criteria and inferred evaluation criteria of intra- and intersubject data. $$^{***}$$ represents the significant difference between the two groups (Wilcoxon’s signed-rank test, $$p<$$ 0.001).

Finally, we asked whether each participant employed individual evaluation criteria, as reflected in their behaviors, and if the inferred evaluation criteria, based on the ErrP-BCI outputs, successfully reflected them. To answer it, we transferred individually calibrated classifiers across participants that predict correct or erroneous trajectories based on the energy efficiency and comfort of the trajectories. Classifiers trained with the behavioral response better modelled their evaluation criteria relative to those trained with ErrP-BCI output (a two-way repeated measures ANOVA revealed the main effect of teaching labels ($F(1, 16)=16.2$, $p=0.001$) (Fig. 6a and Table 1). Furthermore, intra-subject data better predicted individual evaluation criteria relative to inter-subject for both behavior and ErrP conditions (a two-way repeated measures ANOVA revealed the main effect of classifier transfer ($F(1, 16)=19.5$, $p<0.001$)). This shows that evaluation criteria for robot trajectories were customized for each individual, thus intra-subject data better inferred their evaluation criteria than inter-subject condition. Furthermore, we computed the similarities between behavioral and inferred evaluation criteria for each possible pair of subjects without modeling the weighting on the two parameters. Similar to the previous analysis, the higher level of similarity was observed for intra-subject data (0.65 ± 0.04) relative to inter-subject (0.51 ± 0.03) (Fig. 6b), indicating that inferred evaluation criteria successfully reflected individual variation in energy efficiency and comfort (Wilcoxon’s signrank test, $p<0.001$).

**Table 1:** (a) Classification accuracy and (b) the results of the post-hoc comparison.

(a). Classification accuracy				
Dataset	Behavior	Behavior	EEG	EEG
Classifier transfer condition	Intrasubject	Intersubject	Intrasubject	Intersubject
Classification accuracy [%]	92.2 ± 0.74	89.7 ± 0.67	89.9 ± 0.98	88.1 ± 0.89

(b). Post-hoc analysis				
Condition 1	Condition 2	Estimated difference	Standard error	*p*-value
Behavior-Intrasubject	Behavior-Intrasubject	2.51	0.42	$p<$ 0.001
EEG-Intrasubject	EEG-Intrasubject	1.86	0.72	$p =$ 0.02

In summary, our results support the fact that the participants employed individually customized evaluation criteria in the energy efficiency and comfort space to evaluate robot trajectories. This individual customization of the evaluation criteria was successfully reflected in the inferred criteria (intra-subject: $89.9 \pm 0.98$%, inter-subject: $88.1 \pm 0.89$%, $p=0.02$), indicating that ErrPs encode individual weights on “energy efficiency” and “comfort”.

## Discussion

We employed the robot arm as the agent that performed continuous movements while participants monitored its action to evaluate that the robot successfully avoided the obstacle placed in the middle of the path according to one’s evaluation criteria. Robot trajectories were controlled by a dynamical system to generate human-like motion dynamics to facilitate participants’ acquisition of a forward model of the trajectories^48,49^ and the elicitation of ErrPs when trajectories were deemed undesirable. Our results showed that each participant employed individual evaluation criteria, attributed to distinct weighting of energy efficiency and comfort. Furthermore, ErrP-BCI output successfully recreated these individual weighting. This provides novel evidence that ErrP decoding can be used for finer grained inference on the neural correlates underpinning control of reaching movements.

Participants deemed trajectories passing too closely by the obstacle undesirable, and thus used criteria similar to those at play when one is producing similar reaching movements in the presence of obstacles^32,50^. We attribute this observation to the fact that the robot’s dynamics of movement shares similarities with the natural dynamics of human movements. However, to confirm this hypothesis, further works should compare these findings to the use of non-human like dynamics to drive the robot’s movements.

The participants tended to reject the robot’s trajectories that were energy efficient, but offered insufficient comfort (Fig. 4a). Individual differences were most visible at the decision boundary between accepted and rejected trajectories and were indicative of each participant’s unique weighting of the importance of energy efficiency and comfort when judging the trajectories (Fig. 4b). There has been ample evidence that humans’ reaching trajectories are influenced by the presence of obstacles^50–52^, even when these obstacles are not directly in the way^53^. Most of these studies focused on identifying common patterns of movements across individuals and how these movements were influenced by the obstacle’s positioning or height. Other authors have documented that individuals differ in their reaching trajectories when their hand would pass the same static obstacle^5^. These studies have attributed this individual difference to personal preferences and the cost that a person associates with a collision^54^. To our knowledge, no previous investigation has delved into the nature of these costs and how they may vary. Therefore, our study provides fresh insight into the specific costs that individuals use to guide their trajectory planning when faced with obstacles. Our study also revealed that the CNS’s use of two well-established criteria, energy efficiency and comfort, for guiding reaching movements in open space could also explain the planning of reaching movements in the presence of obstacles. We found that the weighting of these two criteria played an important role.

In previous works^55–57^, characterization of the individual evaluation criteria for robot trajectories was carried out by participants physically moving the robots. However, the participants’ view point during physical guidance and subsequent evaluation may vary, thus causing the same robot trajectories to be perceived differently. As an alternative approach to extract individual evaluation criteria for robot actions, our previous work proposed and demonstrated the use of ErrPs combined with IRL^23^. Extending our previous report, the ErrP decoding outputs successfully inferred individual evaluation criteria with high precision (89.9 ± 0. 98%), characterizing individual weighting of energy efficiency and comfort (Fig. 6). These new results now suggest that ErrPs could be used in conjunction with an optimal control approach to infer individual costs used by the CNS to plan movements.

We transferred an individually calibrated decoder between participants to confirm our modeling of individual differences and our modeling of the decision boundary (Fig. 6a). This approach was based on the use of a binary classification model. Although this binary classifier has been commonly used to monitor cognitive processes, it is only a proxy of the true complexity of human decision^58^. Moreover, we considered solely two costs, energy efficiency and comfort, but multiple other factors may be at play.

Previous studies showed that ErrPs encode subjective aspects of performance monitoring such as consciousness^59,60^, confidence^61^ and individual ability to perceive error^62^. In ErrP-based BMI applications, ErrP decoding probabilities were exploited to teach (quasi) optimal policies to external agents^18,23,63,64^. Our study uncovered another aspect of the individual modulations of ErrP that reflects subjective preferences related to motor control during reaching movements (Fig. 5). This novel kind of information, being more informative than a simple probability, could accelerate the teaching process and, critically, incorporate explainability in human-robot interaction. Indeed, as our work shows, optimal control policies acquired through an ErrP-based BMI not only reflect individual preferences for the robots humans interact with, but also are grounded on brain activity. We speculate that this will facilitate the incorporation of neuroprosthesis in our brain schemas as a natural part of our bodies.

## Methods

### Experimental protocol

Seventeen healthy subjects, $24 \pm 2$ years old, participated in the study. All experiments were carried out in accordance with the experimental protocol approved by the EPFL local ethics commission (PB$\_$2017-00295). Informed consent was obtained from all participants who volunteered to perform the experiments. Figure 1 shows the experimental setup.

Participants wore an EEG cap and sat in a chair facing a 7-degree-of-freedom robotic arm (KUKA LWR 4) moving from left to right and back, while avoiding a wine glass located in the middle of the trajectory (Fig. 2). Participants could initiate and interrupt the robot’s motion using a joystick. They were informed of possible collisions between the robot’s end-effector and the wine glass, and were instructed to release the joystick each time they perceived the undesirable robot trajectory (i.e., it risked colliding with the obstacle). Upon the release of the joystick, the robot departed from the trajectory to follow a predefined upward motion, passing above the obstacle until reaching its destination. Participants perceived modification of the ongoing robot trajectories by change in motor sound.

In this setup, error-related EEG activity was superimposed on motor-related EEG activity as participants had to release the joystick to signal their erroneous perception. To verify the null effect of subjects using the joystick on the error-related EEG activity, we recruited an additional group of seven participants in our previous study^23^. The participants performed the task with and without the joystick. In trials without the joystick, the participants reported their preference on the completed trajectories after each trial while the robot was controlled by the experimenter. We confirmed that error-related EEG activity was comparable between with and without the joystick condition. Please refer to our previous study for details^23^.

The robot trajectory was generated by a dynamical system designed for obstacle avoidance^65^. To what extent the trajectory would avoid the obstacle was controlled through two parameters: safety (*s*) and reactivity ($\rho$). These parameters were randomly chosen over the safe region of operation (sampled from homogeneous distributions, $\rho \in [1, 8]$, and $s \in [1.0, 1.5]$) for each trial, allowing the possibility for the arm to hit the obstacle. Collisions between the robot’s end effector and the obstacle occurred for eight subjects, $4 \pm 1$ times per participant.

A trial was considered complete once the robot reached the opposite side of the table with respect to the initial position. Trials were labeled as erroneous or correct depending on whether the participant released the joystick or not, respectively. Each participant completed approximately 400 trials in four runs. The experiment lasted about two hours. Participants rested a few minutes between runs. The average error rate among subjects was 27 ± 7%. In order to represent examples of single-subject individual evaluation criteria and their individual variations, we chose two specific participants who had the maximum or minimum rate of rejecting the trajectories. Specifically, the participant with the highest probability of rejecting the trajectories was considered risk-averse, while the one with the smallest probability was considered risk-prone.

### Robot controller for obstacle avoidance

The robot’s trajectories were controlled in Carthesian space by modulating the trajectory of the robot’s end-effector. Following the established approach^65^, the robot’s motion is driven by a nominal first order dynamical system $\dot{x}=f(x)$, where $x\in \mathbb {R}^3$ represents the state of the robot’s end-effector, which dictates the behavior of the robot in the absence of the obstacle. Close to the obstacle, the dynamics is modulated as follows (Figs. 1 and 2):

1 $$\begin{aligned} \dot{x} = M(x;\rho ,\Gamma (x))f(x) \end{aligned}$$

The columns of *M*(*x*) are composed of a basis of the space, with the first column aligned with the normal *n*(*x*) to the surface to the obstacle.

2 $$\begin{aligned} M(x)= & {} E(x) D(x) E(x)^{-1} \end{aligned}$$

3 $$\begin{aligned} E (x)= & {} \left[ n(x) \;\; e_1(x) \;\;.. \;\; e_{d-1}(x) \right] \end{aligned}$$

where $e_{(\cdot )}$ form an orthonormal basis of the tangent plane to the obstacle. Such a decomposition exists, for convex-shaped obstacles. The approach assumes that a measure of the distance from the obstacle is given by $\Gamma (x)$, where the isoline $\Gamma (x)=1$ corresponds to the obstacle’s boundary. *D*(*x*) is a diagonal matrix, of which the elements increase or decrease the velocity along the direction set by *E*. We set the eigenvalues to

4 $$\begin{aligned} D(x)= & {} {\textbf {diag}} \left( \lambda ^1(x), \, \ldots , \lambda ^d(x) \right) \end{aligned}$$

5 $$\begin{aligned} \lambda ^1(x)= & {} 1 - \frac{1}{{\Gamma (x;s})^{1/\rho }} \qquad \lambda ^d(x) = 1 + \frac{1}{{\Gamma (x;s)}^{1/\rho }}. \end{aligned}$$

The deflection is parameterized by the reactivity factor $\rho$, which controls for how early the robot starts departing from its nominal trajectory. The safety margin *s* around the obstacle is determined by the isoline of value $\frac{1}{\Gamma (x)}$. Specifically:

- The reactivity factor $\rho$ controls the magnitude of the modulation. Increasing $\rho$ has the effect of stronger modulation of the vector field at every point, and hence a earlier response of the robot to the presence of obstacles.
- $\Gamma (x)= \sum \limits _{i=1}^d \left( \frac{x_i}{a_i} \right) ^{2p_i}$ where $a \in \mathbb {R}^d$ and $p \in \mathbb {Z}_+^d$ are component-wise parameters that represent the scale and shape of the object, respectively. A desired safety margin *s* can be achieved with a simple scalar multiplier for *a*. This increases the virtual size of the object, resulting in a larger margin around the object.

In order to relate the parameters of the control system generating the robot trajectories to energy efficiency and comfort, we calculated, respectively, the total distance travelled, given by:

6 $$\begin{aligned} \sum _{t=0} \sqrt{(x_t - x_0)^2} \end{aligned}$$

and the minimum distance between the end-effector and obstacle, given by,

7 $$\begin{aligned} \min (\sqrt{(x_t - x_{obstaxle}})^2). \end{aligned}$$

Since, for erroneous trajectories, the robot did not complete the entire initial trajectory, we used the expected trajectory the robot would have traveled through for this set of parameters for our analysis, assuming that participants used a forward model of the entire trajectory to evaluate if the trajectory was deemed erroneous or not.

### EEG acquisition and processing

EEG signals were recorded from sixteen active electrodes located at Fz, FC3, FC1, FCz, FC2, FC4, C3, C1, Cz, C2, C4, CP3, CP1, CPz, CP2 and CP4 (10/10 international system). EOG signals were also collected from three active electrodes at above the nasion and below the outer canthi of the eyes^66^. The ground electrode was placed on the forehead (AFz) and the reference electrode was placed on the left earlobe. These signals were sampled at 512 Hz and power-line notch filtered at 50 Hz. Participants were asked to refrain from excessive eye movements and blinks when the robot was moving. A screening period of around 50 trials was performed to get them used to the task and visually inspect the EEG signals.The channel CPz of S11 was removed and spherically interpolated because it was identified as a contaminated channel during visual inspection.

Participants underwent 90 s of calibration period in which they were asked to perform the following eye movements without moving their head; (i) clockwise and counterclockwise rolling of eye balls for 30 s, (ii) horizontal and vertical eye movements for 30 s, and (iii) repeated eye blinks for 30 s. These data were used to calculate the coefficients to remove EOG contamination from EEG signals based on an autocovariance matrix^66^.

A second-order high-pass non-causal Butterworth filter was applied with the cut-off frequency of 1 Hz to remove the baseline drift. EEG signals were then epoched with a time window of [-0.2 0.6] s around the onset. We used different onsets for correct and erroneous trials, as the participant did not provide a behavioral response in correct trials. For erroneous trials, the onset was defined as the time when the subjects released the joystick. For the correct trials, the onset was chosen as the individually averaged release time in erroneous trials during the experiment relative to the initiation of robot trajectories; i.e., 1.08 ± 0.14 s (mean ± std). Erroneous trials with a reaction time below 0.5 s or above half of the trial length, i.e., when the robot overtook the obstacle, were removed from the subsequent analysis, since the evaluation was too early or too late with respect to the onset of the trajectory, respectively. This process removed $16.3 \pm 21.7$ trials ($3.54 \pm 4.3\%$ of the total data, mean ± std.).

Artifactual epochs were identified and rejected using the established method based on channel-wise joint log probabilities^67,68^. To estimate the relative probability of each trial for each channel, the observed probability density function of the EEG amplitude values ($D_c$) was calculated for all trials for each channel *c*. Each data-point sample was associated with a probability. Based on this associated probability, the joint log probability $J_c(i)$ was computed for each trial *i* and channel *c* by the following equation:

8 $$\begin{aligned} J_c(i) = -\log (\prod _{x \in A_i} p_{D_c}(x)) \end{aligned}$$

Trials with a deviant probability of at least one channel were removed from subsequent analysis. The criterion was a probability of occurrence above three times the standard deviation from the mean. On average, $23.3 \pm 8.5$ trials were considered spurious ($5.5 \pm 2.1\%$ of total data, mean ± std.).

### Time-frequency analysis

The S-transform was used to decompose the EEG signals into time and frequency domain. The S-transform overcomes the minimal resolution of the short-time Fourier transform and the deficiency of phase recommendation in the Wavelet transform^21,34,69,70^. For localizing the complex Fourier signal, the S-transform uses a window which is Gaussian in nature whose height and width are controlled by frequency. S-transform $S_x(\tau , f)$ of EEG signals *x*(*t*) is defined as follows:

9 $$\begin{aligned} W_x(\tau , d)= & {} \int _{-\infty }^{\infty } x(t) w(t-\tau , d) dt \end{aligned}$$

10 $$\begin{aligned} S_x(\tau , f)= & {} \exp ^{i2\pi f \tau } W_x(\tau , d) \end{aligned}$$

where $W_x(\tau ,d)$ is the wavelet transform of signal *x*(*t*) and the mother wavelet *w*(*t*, *f*) is chosen as follows:

11 $$\begin{aligned} w(t, f)= & {} \frac{|f|}{\sqrt{2\pi }} \exp ^{-\frac{t^2f^2}{2}} \exp ^{-j2 \pi ft} dt \end{aligned}$$

12 $$\begin{aligned} S(\tau , f)= & {} \frac{|f|}{\sqrt{2\pi }} \int _{-\infty }^{+\infty } \exp ^{-\frac{-(\tau - t)^2f^2}{2}} \exp ^{-2i\pi ft} x(t) dt. \end{aligned}$$

The average spectrum of erroneous trials (*ERSP*(*f*, *t*)) was characterized relative to that of correct trials averaged within the time window of [-0.1 0.4] s, $\mu (f)$, as follows^71,72^:

13 $$\begin{aligned} ERSP(f, t) = 10\log _{10}(\frac{ERS(f,t)}{\mu (f)}). \end{aligned}$$

The time and frequency range were set to [-0.5 1.0] s and [1 30] Hz, respectively. Relative power change at each channel location was also calculated by averaging over ERSP from -0.1 to 0.4 s.

### Decoding of ErrPs

EEG signals were low-pass filtered at 16 Hz with a non-causal second-order Butterworth filter (Fig. 3a and b)^17,73,74^, and segmented into epochs within a time window of [-0.1 0.4] s with respect to the onset of events^75^. To enhance signal-to-noise ratio (SNR), we applied a Canonical Correlation Analysis (CCA) based spatial filter and used the first three components for subsequent analysis^76–78^. Then the amplitudes downsampled at 64 Hz and Welch’s Power Spectral Densities (PSDs) from 4 to 16 Hz with steps of 2 Hz were extracted as features. For each trial, we used these two types of features (i.e., amplitude and PSD of the first three CCA components, Fig. S3) because, although their combination did not yield a higher decoding performance than each type separately during ten-fold cross validation (amplitude: 0.85 ± 0.094, PSD: $0.62 \pm 0.069$, combined: $0.86 \pm 0.088$ (mean ± SD)), they have been shown to be superior in other studies^21,23,62,79^. All computed features were concatenated and normalized within the range of [0, 1]. From this feature vector ${\textbf {x}}$, we computed the posterior probability of having detected an error, $p(error|{\textbf {x}})$ using a linear discriminant analysis (LDA) and a ten-fold cross-validation.

14 $$\begin{aligned} p(error|{\textbf {x}}) = \frac{1}{1+\exp ^{-(\textbf{w}'\textbf{x}+b)}} \end{aligned}$$

Robot trajectories were inferred as correct if the probability was below 0.5, while considered erroneous if the probability was above 0.5.

### Visualizing individual evaluation criteria

Visualization of the evaluation criteria was performed by creating 40 linearly separated bins for *energy efficiency* and *comfort*. The min-max ranges were [5.87 5.93] and [0.99 3.10], respectively, based on the simulated robot trajectories. For each trajectory, we associated a Boolean variable to account for the presence of a behavioral response, namely whether the subject had released or not the joystick to interrupt the trajectory. Similarly, each trajectory was associated with the probability of ErrP detection, represented as a scalar value between 0 and 1.

To investigate which of the two parameters, energy efficiency or comfort, better predicts the behavioral response as well as ErrP-BCI output, we performed a logistic regression with only one of the two parameters. The classification accuracy was calculated through a ten-fold cross-validation.

### Assessment of individual evaluation criteria

In order to assess our hypothesis that participants employed individual evaluation criteria when monitoring robot trajectories, we transferred an individually calibrated classifier across participants. Classifiers were trained to predict whether a trajectory was deemed correct or erroneous (class label) based on the measure of energy efficiency and comfort. To determine whether an individual classifier generalized across participants, we contrasted each individual classifier’s accuracy at predicting the intra-subject data versus the inter-subject data. Had all subjects employed the same evaluation criteria, regardless of whose classifier was used to predict the evaluation outcome, inter-subject and intra-subject classification performances should be similar. To avoid overfitting on intra-subject data, performance was evaluated by a ten-fold cross-validation, using a training set to testing set ratio of 90 to 10 $\%$.

This analysis was repeated using the prediction of the ErrP decoder as binary output (class label) relative to the theoretical decision threshold (i.e., 0.5). The aim of the analysis is to measure the quality of the inferred evaluation criteria, thus the predicted ErrP output was compared against the behavioral response. The classification accuracy is 100% if the classifier trained with the ErrP-BCI output successfully predicted the behavioral responses of the participants for observed pairs of energy efficiency and comfort. We contrasted classification performance between the intra- and inter-subject to evaluate variability of prediction across participants.

In both analyses, we evaluated the classification performance using a two-by-two conditional matrix. One condition is classifier transfer, i.e., intra- or inter-subject, while another is the type of teaching labels, i.e., behavioral response or decoder output. We used a two-way repeated measures analysis of variance (ANOVA) to evaluate the variability of classification performance over the two-by-two conditions.

To further determine if inferred individual preferences by ErrP detection were customized for each participant and for each pair of energy efficiency and comfort, we computed the Pearson correlation coefficients between behavioral and inferred evaluation criteria for intra- and inter-subject data. Computed correlation coefficients of the two conditions were then assessed by Wilcoxon’s signed rank test.

### Supplementary Information

Supplementary Information.

## Data Availability

The datasets generated and/or analyzed during the current study are available in the Zenodo repository, https://zenodo.org/record/3627015.
