# Supplementary material for: Inferring individual evaluation criteria for reaching trajectories with obstacle avoidance from EEG signals
Source: Sci Rep. 2023 Nov 17;13:20163. doi: 10.1038/s41598-023-47136-2 (PMC10656489; doi:10.1038/s41598-023-47136-2)
Supplement: Supplementary file 1 — Supplementary Information. [file 41598_2023_47136_MOESM1_ESM.pdf]

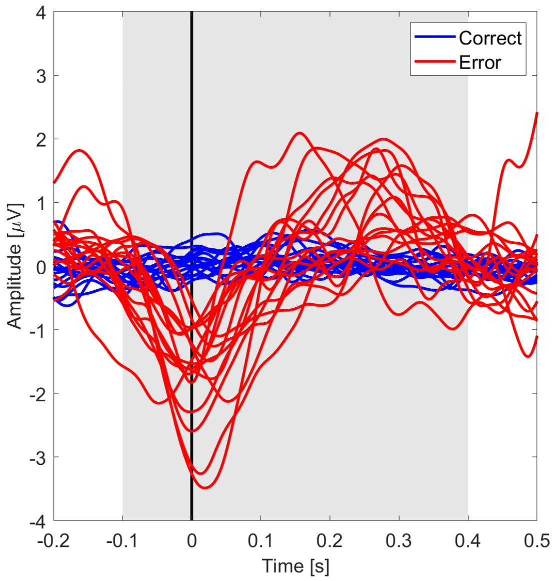

Figure S 1: Individual grand-averaged event-related potentials of correct and erroneous trials at FCz. 0 s in the x-axis represents the onset of release for erroneous trials, while it corresponds to the individual averaged release time for correct trials. The gray shaded area corresponds to the time window used for decoding analysis.

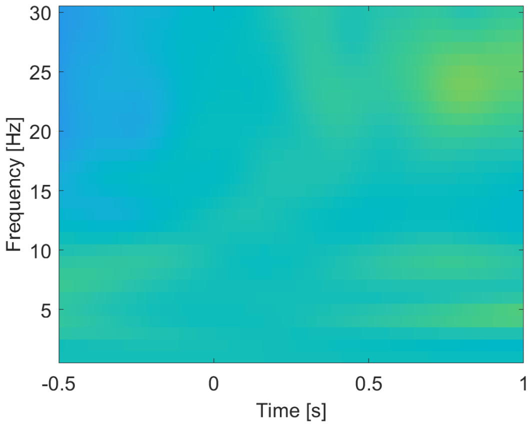

(a) ERSP at FCz

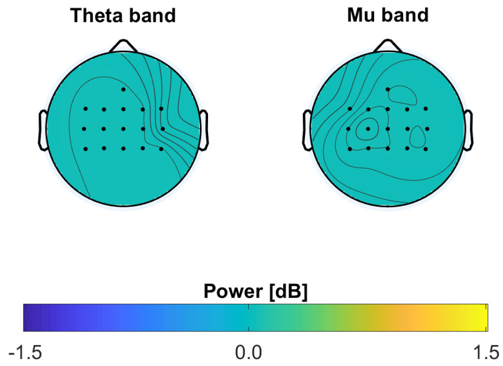

(b) Theta and Mu band power

Figure S 2: Supplementary Electrophysiological results. (a) Time-frequency representation of event-related spectral perturbation (ERSP) in correct trials at FCz within the time window of [-0.5 1] s with respect to individual average time to release the joystick in erroneous trials. (b) Theta band ([4 8] Hz) and mu band ([8 12] Hz) spectral power of all channels in correct trials.

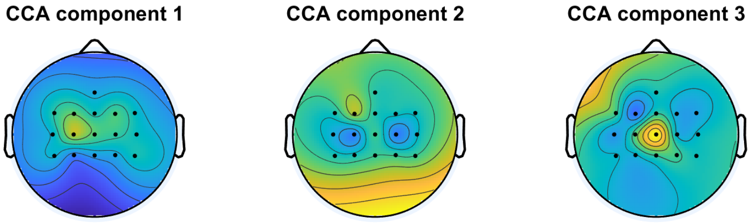

Figure S 3: Example weights of the first three spatial filters (corresponding to the first three columns of the spatial filter matrix) when using CCA for spatial filtering.
